# Developmental Trends of Visual Processing of Letters and Objects Using Naming Speed Tasks

**DOI:** 10.3389/fnhum.2020.562712

**Published:** 2020-12-10

**Authors:** Kaitlyn Easson, Noor Z. Al Dahhan, Donald C. Brien, John R. Kirby, Douglas P. Munoz

**Affiliations:** ^1^Department of Biomedical and Molecular Sciences, Queen's University, Kingston, ON, Canada; ^2^Centre for Neuroscience Studies, Queen's University, Kingston, ON, Canada; ^3^Faculty of Education, Queen's University, Kingston, ON, Canada

**Keywords:** naming speed, eye tracking, orthographic processing, phonological processing, development

## Abstract

Studying the typical development of reading is key to understanding the precise deficits that underlie reading disabilities. An important correlate of efficient reading is the speed of naming arrays of simple stimuli such as letters and pictures. In this cross-sectional study, we examined developmental changes in visual processing that occurs during letter and object naming from childhood to early adulthood in terms of behavioral task efficiency, associated articulation and eye movement parameters, and the coordination between them, as measured by eye-voice span in both the spatial and temporal domains. We used naming speed (NS) tasks, in which participants were required to name sets of letters or simple objects as quickly and as accurately as possible. Single stimulus manipulations were made to these tasks to make the stimuli either more visually and/or phonologically similar to one another in order to examine how these manipulations affected task performance and the coordination between speech and eye movements. Across development there was an increased efficiency in speech and eye movement performance and their coordination in both the spatial and temporal domains. Furthermore, manipulations to the phonological and visual similarity of specific letter and object stimuli revealed that orthographic processing played a greater role than phonological processing in performance, with the contribution of phonological processing diminishing across development. This comprehensive typical developmental trajectory provides a benchmark for clinical populations to elucidate the nature of the cognitive dysfunction underlying reading difficulties.

## Introduction

Reading is a critical skill for a child's overall developmental trajectory (Norton and Wolf, 2012). To better understand the development of this complex skill, it is important to examine the typical development of the cognitive, visual, and articulatory processing underlying reading. These key component processes can be studied using tests of naming speed (NS), the speed and accuracy with which individuals can name familiar alphanumeric stimuli, such as letters or numbers, or non-alphanumeric stimuli, such as objects or colors, presented in a visual array (Wolf and Bowers, 1999). This simple paradigm is a valuable tool for studying many of the numerous cognitive, articulatory, and oculomotor processes involved in reading and the efficiency of the underlying timing mechanisms that connect them (see Wolf et al., 2000; Cutting and Denckla, 2001; Al Dahhan et al., 2016, in press). Across development, NS has been found to be a strong predictor of reading ability (Kirby et al., 2003; Arnell et al., 2009), with the predictive power of alphanumeric NS being greater than that of non-alphanumeric NS (Wolf et al., 1986; Cronin and Carver, 1998; Compton, 2003; Bowey et al., 2005; Araújo et al., 2015). Although most studies of NS focus on alphanumeric NS, particularly letter NS, non-alphanumeric NS tasks remain a useful tool for young children who have not yet mastered letters and numbers (Kirby et al., 2003; Lervåg and Hulme, 2009).

Two key cognitive processes shared by reading and NS are phonological processing and orthographic processing. However, there has been disagreement as to which of these processes makes the greater contribution to NS, by forming the bulk of the cognitive processing that underlies NS. While one theory proposes that NS reflects the automaticity of phonological processing (Torgesen et al., 1994, 1997), the second suggests that NS is instead an indication of the automaticity of recognition of visual symbols and orthographic processing (Bowers and Wolf, 1993; Bowers, 1995). To test these two hypotheses, NS task stimuli can be manipulated to increase their phonological and/or orthographic similarity (Compton, 2003). If NS performance relies primarily on phonological processing, increasing the phonological difficulty of a NS task by selecting stimuli whose names rhyme with one another should impair task performance. However, if NS relies primarily on orthographic processing, increasing orthographic difficulty by selecting stimuli that are visually similar to one another should impair performance. Based on these assumptions, letter NS tasks have previously been employed to identify orthographic processing as the main cognitive process underlying NS task performance in both adults and young children (Al Dahhan et al., 2014, 2017).

In addition to measuring the speed and accuracy of naming, insight into the specific articulatory and oculomotor mechanisms underlying NS can be obtained by studying participants' articulations and eye movements (Hyona and Olson, 1995; Rayner, 1997; Georgiou et al., 2006, 2008; Kirby et al., 2010). Naming time can be divided into articulation time, the time spent pronouncing stimulus names, and pause time, the time between two sequential articulations (Hulme et al., 1999; Neuhaus et al., 2001; Georgiou et al., 2006, 2008). Eye movements can be analyzed to measure the duration of fixations on stimuli, the number of forward saccades, and backward saccades, called regressions (Rayner, 1997). Negative correlations have been found between NS efficiency, referring to the number of items correctly named per second, and these various articulatory components and eye movement measures (Al Dahhan et al., 2017), indicating that shorter pause and articulation times, briefer fixations, and lower saccade and regression counts are markers of improved task performance. Of these constructs, pause time and fixation duration were especially predictive of NS efficiency (Al Dahhan et al., 2017).

Performance on NS tasks can also be examined by analyzing participants' eye-voice span (EVS), which describes how far the eyes are ahead of the voice during oral reading (Buswell, 1922), thus providing insight into the coordination between eye movements and articulations. EVS can be calculated in both spatial and temporal domains. *Spatial EVS* refers to the number of items between the currently-fixated item and the currently-articulated item (Buswell, 1922). Spatial EVS is thought to be related to the updating of the phonological loop of the working memory store, as the visual input that has been translated to a phonological code is held in working memory until it can be articulated (Baddeley and Hitch, 1974; Laubrock and Kliegl, 2015). Spatial EVS has been shown to be longer on NS tasks for typical readers as compared to readers with dyslexia (Pan et al., 2013), as the latter group may struggle with the grapheme-to-phoneme conversion, thus slowing the updating of the working memory buffer.

In contrast, *temporal EVS*, also referred to as naming latency, refers to the time between the first fixation on an item and the onset of the articulation of that item (Inhoff et al., 2011). Temporal EVS provides a measure of the efficiency of all stages of cognitive processing and articulatory planning required to identify and prepare to articulate the name of the stimulus (Jones et al., 2013). Accordingly, dyslexic readers have been found to have longer temporal EVSs than typical readers (Jones et al., 2013), indicating that overall cognitive and articulatory processing efficiency is decreased in dyslexic readers. Thus, examination of developmental trends in both spatial and temporal EVS has the potential to provide key insight into how the processes required for the updating of the working memory buffer and overall cognitive and articulatory processing speed, respectively, during reading evolve across the lifespan.

In this study, we describe the typical developmental trajectory for alphanumeric and non-alphanumeric NS from childhood to early adulthood in terms of NS efficiency, associated articulation and eye movement parameters, and the coordination between them, as measured by EVS in both the spatial and temporal domains. To accomplish this, we administer letter and object NS tasks containing stimuli with varying degrees of phonological and visual similarity to typically developing Grade 2, Grade 4, Grade 7/8, and undergraduate students. We hypothesize that older participants will perform better on NS tasks than younger readers, characterized by higher NS efficiency accompanied by lower pause and articulation times, fixation duration, saccade and regression counts, and temporal EVS, and higher spatial EVS. As a result of the predominant role of orthographic processing during letter NS tasks, we hypothesize that increased visual similarity will impair NS task performance across groups, with a lower impact of increased phonological similarity in older readers, accompanying the shift from phonological processing-dependent phonetic reading to orthographic processing-dependent word-recognition reading (Ehri and McCormick, 1998). Furthermore, we predict that performance will be more efficient on letter NS tasks than object NS tasks due to participants' greater exposure to letters and reliance on automated letter recognition for reading in daily life, as well as minor contributions related to the smaller set size and unambiguous names of letters as compared to objects. Elucidating developmental trends with respect to these NS constructs will provide insight into how key cognitive, oculomotor, and articulatory processes required for reading evolve over the course of typical development.

## Materials and Methods

### Participants

Four groups of healthy participants with no underlying neurological conditions participated in this study: Grade 2 students (*n* = 13, 6 males, ages 7.2–8.1 years, age *M* = 7.7 years, age *SD* = 0.3 years), Grade 4 students (*n* = 14, 7 males, ages 9.3–10.2 years, age *M* = 9.7 years, age *SD* = 0.3 years), Grade 7/8 students (*n* = 21, 8 males, ages 12.2–14.0 years, age *M* = 13.4 years, age *SD* = 0.6 years), and undergraduate students (*n* = 20, 10 males, ages 20.8–22.5 years, age *M* = 21.3 years, age *SD* = 0.3 years). These age groups were selected to sample the NS task performance of younger readers still undergoing reading and literacy instruction at the primary, junior, and intermediate divisions of elementary school and to compare the performance of these developing readers to an older group of skilled readers pursuing advanced education. Participants were recruited from Queen's University and the greater Kingston, Ontario community. Informed consent was provided from participants aged 18 years or older, and from legal guardians for participants younger than 18 years prior to testing.

### Naming Speed Measures

**Figure 1:**
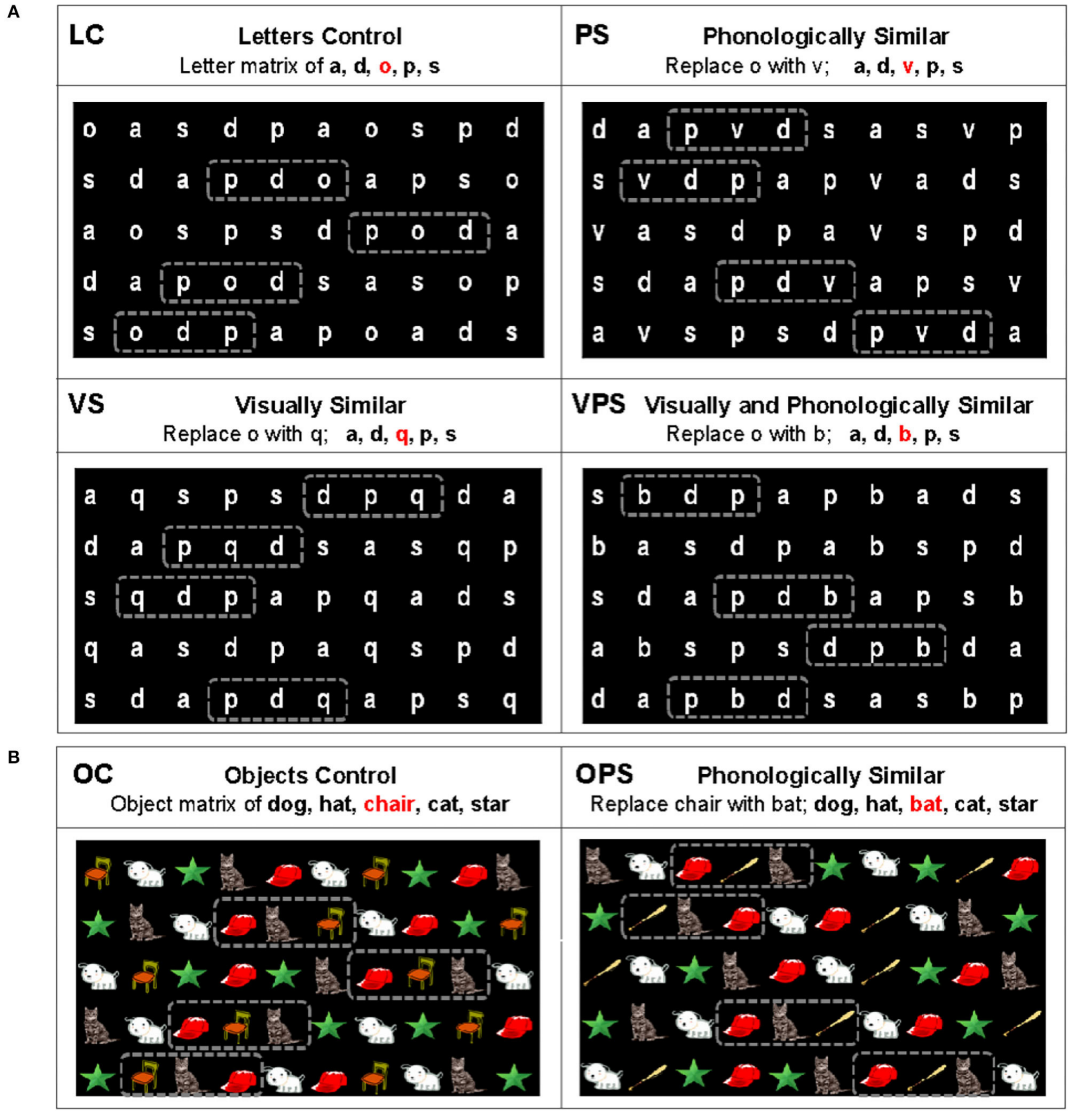
Naming speed (NS) stimuli. **(A)** Letter NS tasks. The letter control (LC) task was developed by Denckla and Rudel (1976), while the phonologically similar (PS), visually similar (VS), and visually and phonologically similar (VPS) tasks were developed by Compton (2003). **(B)** Object NS tasks. The control (OC) task was developed by Denckla and Rudel (1976). Dashed boxes indicate regions where stimuli became similar to one another.

Four letter NS tasks, with two trials per task, were administered, including the original letter NS task developed by Denckla and Rudel (1976) and three variations by Compton (2003) designed to increase the phonological and/or visual similarity of the letters used in the task (Figure 1A). The letter control (LC) task involved a matrix of the letters *a, d, o, p*, and *s*. In the phonologically similar (PS) task, *o* was replaced with *v*; in the visually similar (VS) task, *o* was replaced with *q*; and in the visually and phonologically similar (VPS) task, *o* was replaced with *b*. Two object NS tasks, with two trials per task, were administered (Figure 1B), including the control (OC) task developed by Denckla and Rudel (1976) based on line drawings of *dog, hat, chair, cat*, and *star* and one variation of this task designed to increase phonological similarity (OPS; *chair* replaced with *bat*). In each NS task, 50 letters/objects were presented simultaneously with 10 repetitions of the five letters/objects arranged semi-randomly in five rows of 10 letters/objects each. Participants were instructed to name all the letters/objects out loud as quickly and accurately as possible from left to right and top to bottom, while their articulations and eye movements were recorded. Scores were averaged between the two trials for each task for each measure to compute a single score.

Before the beginning of the tasks, two practice trials were administered for both the letter NS tasks and the object NS tasks. In the first practice trial for the letter NS tasks, participants were asked to name the eight letters to be used to ensure their familiarity with these letters, and in the second practice trial, they were presented with a practice letter NS task of four rows of five letters each to ensure their comprehension of the task instructions. Comparable practice trials were also administered for the object NS tasks, requiring participants to first name the six objects to be used and then complete a practice object NS task of two rows of five objects each.

### Visual Display, Eye Tracking, and Articulatory Recordings

Eye position was recorded using an Eyelink 1000 heads-free eye tracking system (SR Research Ltd., Mississauga, ON, Canada). Participants placed their heads on an adjustable chinrest/headrest and a microphone (Audio Technica, Tokyo, Japan; M-Audio, Cumberland, RI, USA) was positioned near the participants' mouths to record verbal responses. An ASIO compatibility card with 10 ms latency buffer was used. This set up allowed for millisecond accurate synchronization between audio recording and eye tracking. A 17-inch LCD monitor (Resolution 1,280 × 1,024) equipped with an infrared camera for eye tracking was positioned 60 cm from participants' right eye. Although viewing of the display was binocular, all recordings and calibrations were done monocularly based on the right eye. The position of the right pupil was digitized in both the vertical and horizontal axes at a sampling rate of 500 Hz. Eye position was first calibrated using nine randomly presented target locations on the screen: eight peripheral and one central. This process was then repeated to validate the calibration. During the 9-point calibration, a cut-off of 1.0° for average error and 1.5° for maximum error was used. All participants had an average error of 0.5°.

Each NS trial began with the presentation of a central white fixation point to orient each participant to a common location and to check for calibration drift, ensuring that the average error remained <2°. The fixation point then disappeared and the NS array appeared. In both the letter and the object NS tasks, there was a 2.98° viewing distance between each item and a 1.85° viewing distance between each row. The letters in the letter NS tasks were printed in white using size 60 Angsana New font on a black background. The objects in the object NS tasks were presented in color on a black background, with dimensions of 2.30° by 2.02°. After the last item in a trial was named, the array disappeared and was replaced by the fixation point before the presentation of the array for the next trial. Trials were manually advanced by the experimenter and no feedback was given on performance.

### Data Analysis

Eye movements and articulations were analyzed using a combination of built in saccade detection tools from SR Research and custom MATLAB software to mark articulations and compute more complicated measures.

Extraction of data from the audio files was accomplished using a revised protocol based on a previously published method (Georgiou et al., 2006), designed to remove background noise and normalize the volume of the audio recordings across participants (also see, Al Dahhan et al., 2017, 2020). In detail, for each wave file (sampling rate of 44.1 kHz) of audio recording, we performed a simple normalization algorithm to extract absolute amplitude. First, taking the absolute value of the waveform, we removed any extreme loud spikes using a threshold of 7 standard deviations above the mean amplitude, and then normalized to the maximum amplitude. We then used a rectangular box filter of 200 sample points to remove high frequency noise. Empirically, we found that this produced a clean waveform, but some loud spikes remained so we repeated the spike removal at a threshold of 4 standard deviations above the mean. After re-normalizing, a clean wave form of articulation amplitude was produced. To mark the onset and offset of articulations, we applied a simple threshold filter of 15% of the normalized amplitude. The amplitude had to remain above this threshold for 2,000 sample points to be counted as an articulation, in order to avoid short spurious noises being falsely detected. The sound files were separated into pauses and articulations based on this automated method. This automated threshold also agreed with our empirical segmentation upon listening to each articulation.

Some manual segmentation of articulations was also required and a custom software interface in MATLAB was used for this process. This interface displayed the articulations, the corresponding saccades over the letters, and the onset/offset times for articulations. It allowed for further manual annotation. Firstly, some participants would slur articulations or not pause at all between articulations. We manually split these articulations where the amplitude would dip to a minimum (but still above threshold) between these continuous utterances. Pause time would be 0 between these articulations. Secondly, we manually removed spurious articulations such as unrelated talking, coughing, or other utterances. Third, very rarely we would manipulate the end points of an articulation if some other noise interfered with proper detection via the automated method. Overall, we found that little manual intervention was required in most cases and every effort was made to rely on objective, automated marking.

Further, articulations were manually scored to calculate an error score from the participants' responses, corresponding to the number of naming errors made per trial. NS efficiency was then calculated by dividing the number of items named correctly in a trial by the total time spent naming items on that trial. Pause time for each trial was calculated as the mean of the pause times between two correctly identified items, and articulation time was calculated as the mean of the articulation times for correctly identified items. These parameters were calculated after removing the pauses and articulations associated with task errors. Specifically, incorrect articulations and their bordering pauses were removed from the data. If participants corrected themselves following a naming error, the incorrect articulation and the bordering pauses were removed. If participants skipped an item, the pause time between the articulations of the two neighboring items, as well as the articulation of the item following the skipped item, were removed.

Eye movements were analyzed for fixation duration, saccade metrics, and regression counts. An eye movement was counted as a saccade based on the built-in saccade detection algorithm of the SR Research software. Specifically, a saccade was marked when it reached either a threshold velocity of 30°/s or a threshold acceleration of 8,000°/s^2^. We used these start and end points in all subsequent analysis. Amplitude was calculated as the Euclidean distance in degrees of visual angle between these end points. Fixations were calculated as the time intervals between saccades, and the fixation duration was calculated for each trial by averaging the length of all the fixations made in the trial. Regressions were defined as leftward saccades <10° in amplitude and within a horizontal visual angle of 30° in order to exclude leftward eye movements from the end of one line of the array to the beginning of the next line. We found that this method was most discriminating, as reading was rarely a clean horizontal trace along each line and some allowance for off-horizontal regression was required.

**Figure 2:**
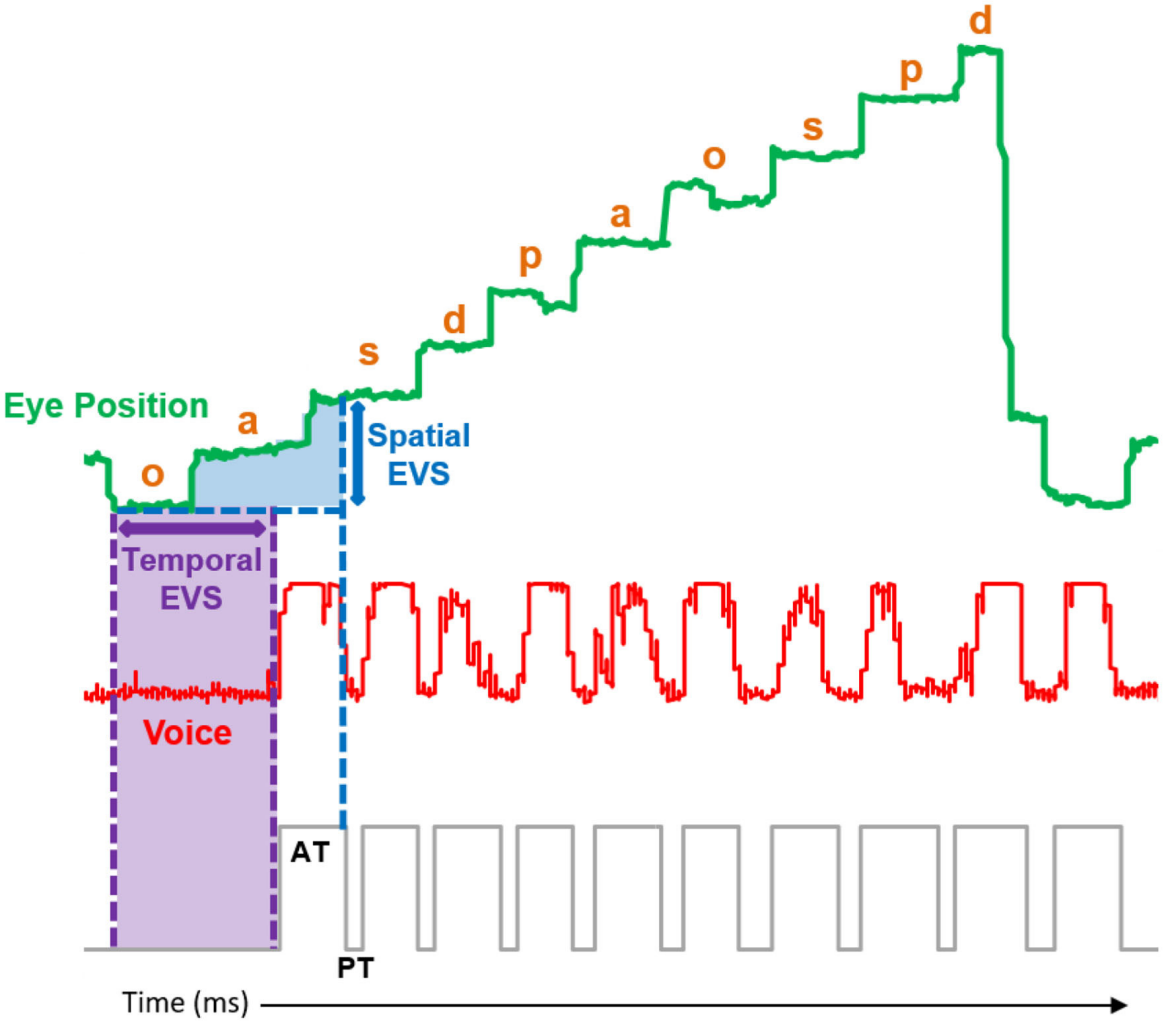
Sample articulation (red trace) and eye position (green trace) collected from a Grade 4 participant performing the letter control naming speed task. Spatial eye-voice span (EVS) was calculated for letter s by determining articulation position (on letter o) at the beginning of the first fixation on letter s (vertical blue arrow). Temporal EVS was calculated for letter o by determining the temporal lag from first fixation on letter o to the beginning of the articulation of letter o (horizontal purple arrow). See text for additional details.

We also analyzed the coordination between the fixation and articulation of each stimulus. We measured the eye-voice span (EVS), the difference between fixation and articulation of a stimulus. Both spatial and temporal EVS were calculated, excluding the leftmost, and rightmost stimulus in each row. *Spatial EVS* was defined as the number of letters or objects that the voice was behind of the eye at the first fixation on a stimulus and expressed as a real number (Figure 2). This was calculated by subtracting the stimulus position being articulated from the eye position at first fixation, and then converting to number of letters as a fraction of the distance between letters (i.e., if a participant was reading A-B-C and was between articulating A and B when they fixated on C, the spatial EVS would be 1.5). *Temporal EVS* was defined as the amount of time between the first fixation of a stimulus and the beginning of the articulation of that stimulus. This calculation was more complicated, as reading was not always a clean horizontal trace along the letters, as mentioned previously. Therefore, we defined fixation of the stimulus as the closest Euclidean distance between the center of the stimulus (letter or object) and the median fixation position. We limited the search to a conservative 3.5 s of previous articulation, to avoid any spurious fixations from earlier or later movements.

Two methods were used to examine both spatial and temporal EVS: (a) region of interest (ROI) -specific EVS, which was calculated by averaging the EVS values of all correctly articulated stimuli within defined ROIs (see dashed boxes in Figure 1) in each trial and (b) EVS outside of the ROIs, which was calculated for each trial by averaging the EVS values of all correctly articulated stimuli outside of the ROIs. These ROIs represent the most challenging sections of each task variation, containing 3–4 adjacent occurrences of the 3 letters or objects that are the most phonologically and/or visually similar. Therefore, the ROI-specific EVS constructs were included to provide a measure that might be more sensitive to stimulus manipulations as compared to EVS calculated outside of the ROIs. Eye movements associated with skipped items or incorrectly named items were removed manually.

## Results

First, we examined how NS task performance changed over the course of development in terms of traditional measures of NS task performance, including NS efficiency, articulatory components, and eye movement measures, and examined the effect of stimulus manipulations on each of these constructs across the developmental trajectory. We then explored how the articulatory components and corresponding eye movement measures were coordinated across development by examining developmental trends in EVS. Finally, we explored the relationships between these behavioral measures and determined which behavioral measures best predicted NS efficiency across development.

### Trends in Traditional Measures of NS Task Performance

**Figure 3:**
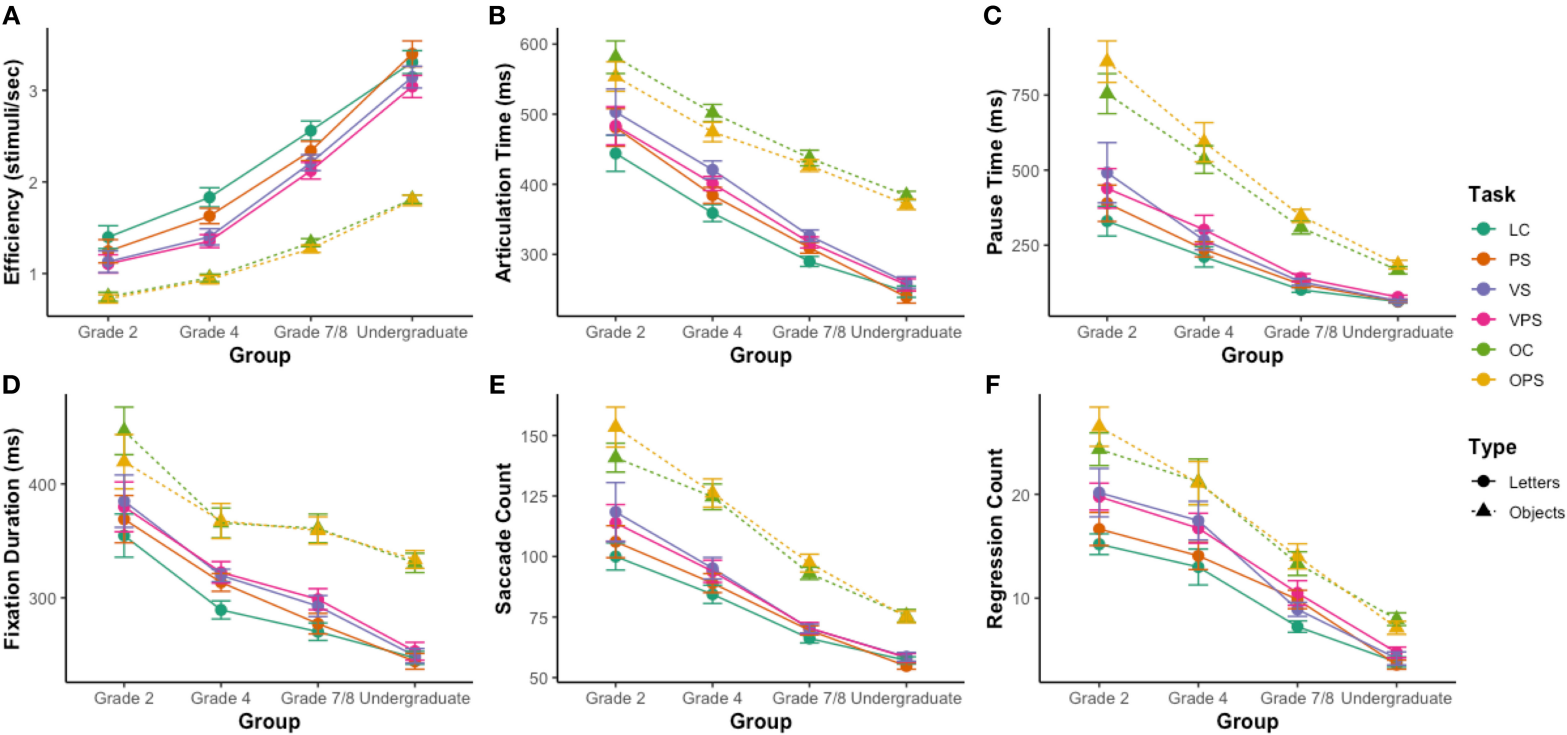
Effect of task version on NS efficiency, NS components, and eye movements across development. **(A)** Efficiency score on the NS tasks. **(B)** Average articulation time per trial. **(C)** Average pause time per trial. **(D)** Average fixation duration. **(E)** Saccade count. **(F)** Regression count. Data for letter NS tasks are denoted with circles and solid lines and data for object NS tasks are denoted with triangles and dashed lines. LC, letter control task; PS, phonologically similar task; VS, visually similar task; VPS, visually and phonologically similar task; OC, object control task; OPS, phonologically similar task. Standard errors are shown.

Figure 3 shows the effect of stimulus manipulations on task performance across development in terms of NS efficiency (Figure 3A), articulation time (Figure 3B), pause time (Figure 3C), fixation duration (Figure 3D), saccade count (Figure 3E), and regression count (Figure 3F). A series of group x all NS tasks mixed analyses of variance, one for each construct, showed significant group effects (all *p*'s < 0.05), significant task effects (all *p*'s < 0.001), and significant group by task interactions (all *p*'s < 0.001) for all measures. The group effects were examined with Bonferroni-corrected *t*-tests using the averaged *z*-scores of the six NS tasks. For NS efficiency and articulation time, there were significant differences between each pair of adjacent age groups, with higher NS efficiency and shorter articulation times for the older groups (Figures 3A,B; all *p*'s < 0.05). Pause time, saccade count, and regression count were all significantly lower for the Grade 7/8 students than the Grade 4 students and for the undergraduate students than the Grade 7/8 students (Figures 3C,E,F; all *p*'s < 0.05), with no significant difference between the Grade 2 and Grade 4 students. Furthermore, fixation duration was significantly lower for Grade 4 students than Grade 2 students and for undergraduate students than Grade 7/8 students (Figure 3D, all *p*'s < 0.05), with a plateau in this developmental decline between Grades 4 and 7/8.

With respect to the task effect, we first investigated the difference in performance during the letter and object NS tasks. On all measures, performance on each of the letter and object NS task versions was significantly correlated (*r* = 0.68–0.98, all *p*'s < 0.01). Based on these significant correlations, composite scores for each of the letter and object NS tasks were computed for each of the constructs, by averaging the raw scores for the four letter NS tasks and the raw scores of the two object NS tasks. Using these composite scores, paired-samples *t*-tests showed that there was a significant difference between the letter and object NS tasks on all measures, with increased efficiency [Figure 3A; *t*_(67)_ = 15.4, *p* < 0.001, *r* = 0.92, *d*_*z*_ = 1.9], shorter articulations [Figure 3B; *t*_(67)_ = −17.4, *p* < 0.001, *r* = 0.84, *d*_*z*_ = −2.1] and pauses [Figure 3C; *t*_(67)_ = −14.4, p < 0.001, r = 0.92, *d*_*z*_ = −1.7], shorter fixations [Figure 3D; *t*_(63)_ = −14.8, *p* < 0.001, *r* = 0.81, *d*_*z*_ = −1.9], and fewer saccades [Figure 3E; *t*_(63)_ = −15.5, *p* < 0.001, *r* = 0.90, *d*_*z*_ = −1.9] and regressions [Figure 3F; *t*_(63)_ = −10.4, *p* < 0.001, *r* = 0.89, *d*_*z*_ = −1.3] on the letter NS tasks. These factors are typically indicative of stronger task performance.

When considering the phonologically and visually similar manipulations, for the letter NS tasks, paired-samples *t*-tests showed that the combined visually and phonologically similar task manipulation (i.e., the VPS condition) had the greatest effect on performance overall, resulting in decreased NS efficiency (Figure 3A), longer articulation times (Figure 3B), pause times (Figure 3C), and fixation durations (Figure 3D), and an increased number of saccades (Figure 3E) and regressions (Figure 3F) relative to the control task (i.e., the LC condition; all *p*'s < 0.001). The phonologically similar manipulation (i.e., the PS condition) and the visually similar manipulation (i.e., the VS condition) both had significant effects on each of these measures of task performance compared to the LC condition (all *p*'s < 0.01). The VS manipulation exerted a greater effect than the PS manipulation on all measures, with statistically significant differences between the two conditions (all *p*'s < 0.05). Notably, the VS manipulation exerted a greater effect than the VPS manipulation on articulation time, as articulation time was significantly longer on the VS condition as compared to the VPS condition (Figure 3B). For the object NS tasks, paired-samples *t*-tests showed that there was a significant difference between the two tasks on NS efficiency (Figure 3A), articulation time (Figure 3B), pause time (Figure 3C), and saccade count (Figure 3E), in which participants were overall less efficient and made shorter articulations, longer pauses, and more saccades on the OPS task than the OC task (all *p*'s < 0.05).

We then examined the significant group by task interactions to determine how the effects of task composition and stimulus manipulations differed across development. First, to determine the contributions of the different developmental trajectories of letter and object NS tasks to the group by task interactions, we repeated the analyses of variance using the average letter and object composite scores. Significant interactions between group and task remained for efficiency, pause time, saccade count, and regression count (all *p*'s < 0.05). Visual inspection of the developmental trends for these constructs reveals that these significant interactions can be attributed to a greater difference between efficiency for letter and object NS tasks in the older groups (Figure 3A), but a greater difference between pause time, saccade count, and regression count for letter and object NS tasks in the younger groups (Figures 3C,E,F). These patterns suggest a greater developmental increase in efficiency for the letter NS tasks and a greater developmental decline in pause time, saccade count, and regression count for the object NS tasks.

Next, to determine the contributions of the phonologically and visually similar manipulations to the significant group by task interactions, we repeated the analyses of variance separately for the four letter NS tasks and the two object NS tasks. Significant group by task interactions remained for all constructs on the letter NS tasks (all *p*'s < 0.01), but for the object NS tasks, there was only a significant interaction for pause time [*F*_(3, 64)_ = 3.37, *p* < 0.05, $\eta_{p}^{2}\;=$ 0.14]. These findings indicate evolving effects of phonological and visual similarity over the course of development. Overall, the effect of these manipulations was smaller for older groups, with a particular decline in the effect of the phonologically similar manipulation across constructs for the letter NS tasks and a specific decline in the effect of the phonologically similar manipulation on pause time for the object NS tasks. Furthermore, while the phonologically similar manipulation of the letter NS task (i.e., the PS condition) had significant effects on efficiency and saccade count in all four groups, it had a protective effect in the undergraduate group, increasing efficiency [Figure 3A; *t*_(19)_ = 2.13, *p* < 0.05, *r* = 0.95, *d*_*z*_ = 0.48] and decreasing saccade count [Figure 3E; *t*_(19)_ = −3.30, *p* < 0.01, *r* = 0.88, *d*_*z*_ = −0.74] relative to the control task (i.e., the LC condition). These factors are typically associated with improved task performance.

### Eye-Voice Span

**Figure 4:**
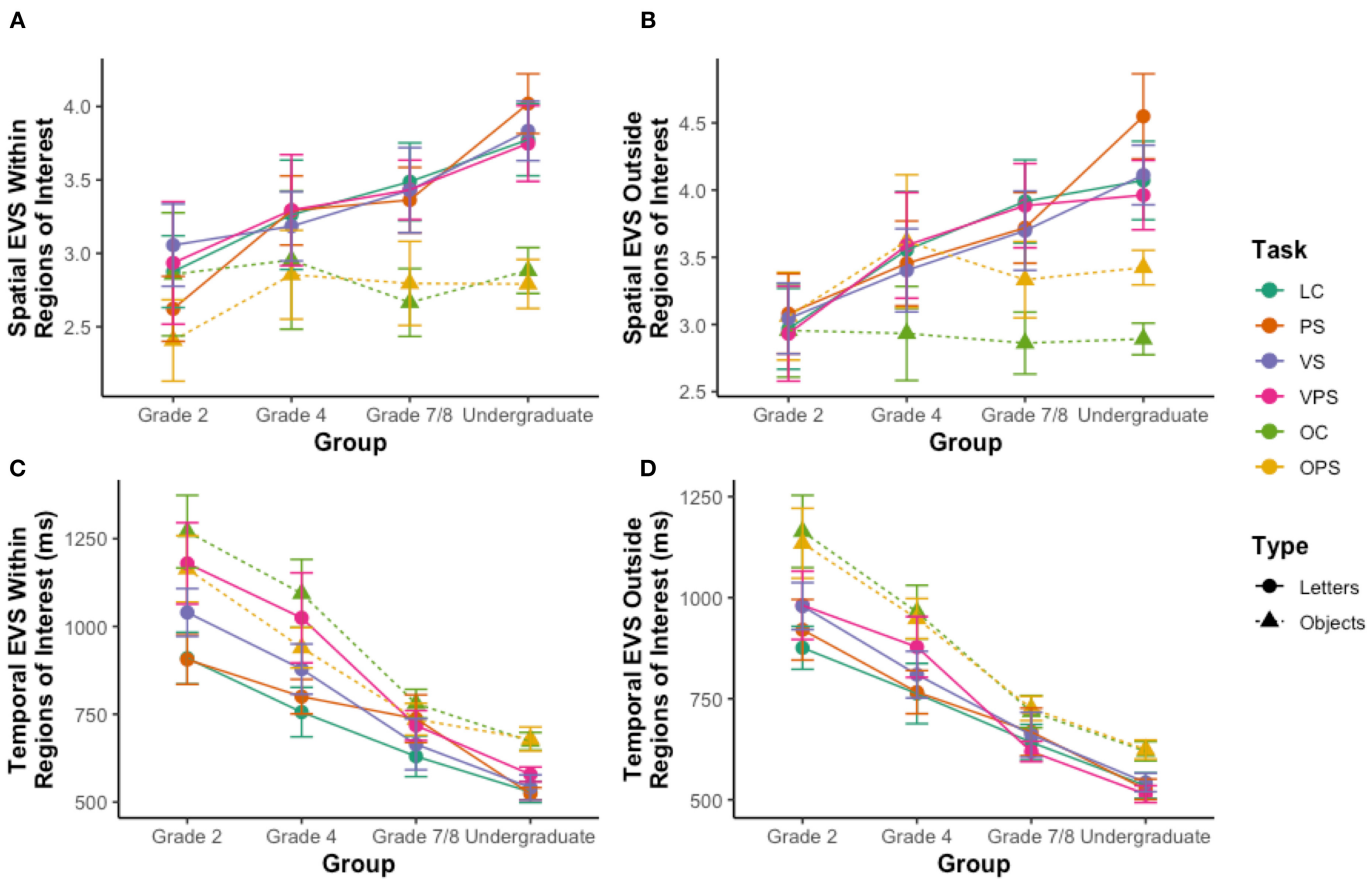
Effect of task version on eye-voice span (EVS) across development. **(A)** Spatial EVS within the regions of interest. **(B)** Spatial EVS outside of the regions of interest. **(C)** Temporal EVS within the regions of interest. **(D)** Temporal EVS outside of the regions of interest. Data for letter NS tasks are denoted with circles and solid lines and data for object NS tasks are denoted with triangles and dashed lines. LC, letter control task; PS, phonologically similar task; VS, visually similar task; VPS, visually and phonologically similar task; OC, object control task; OPS, phonologically similar task. Standard errors are shown.

After confirming the developmental trends that we hypothesized for traditional measures of NS task performance, we next examined the coordination between the articulations and eye movements required for NS by measuring spatial and temporal EVS (see Figure 2 and Methods). Figure 4 shows the effect of stimulus manipulations on spatial EVS for only the specific ROIs illustrated by dotted boxes in Figure 1 (Figure 4A), spatial EVS averaged across stimuli outside of the ROIs (Figure 4B), temporal EVS for the ROIs (Figure 4C), and temporal EVS averaged across stimuli outside of the ROIs (Figure 4D).

A series of group x NS task mixed analyses of variance, one for each EVS construct, showed significant group effects for both temporal EVS measures (all *p*'s < 0.001) but neither spatial EVS measure (all *p*'s > 0.05), significant task effects for all measures (all *p*'s < 0.001), and significant group by task interactions for spatial EVS and temporal EVS outside of the ROIs (all *p*'s < 0.05). With respect to the group effect for the temporal EVS constructs, Bonferroni *post-hoc* tests using the averaged *z*-scores of the four letter NS tasks and the two object NS tasks revealed that the Grade 7/8 students had significantly shorter temporal EVSs than the Grade 4 students and the undergraduate students had significantly shorter temporal EVSs than the Grade 7/8 students (all *p*'s < 0.05), for both temporal EVS constructs.

With respect to the task effects for the four EVS constructs, we first investigated the difference in performance during the letter and object NS tasks. For all EVS constructs, performance on each of the letter and object NS task versions was significantly correlated (*r* = 0.46 to 0.92, all *p*'s < 0.001). Based on these significant correlations, composite scores for the letter and object NS tasks were computed for each of the constructs, by averaging the raw scores for the four letter NS tasks and the raw scores for the two object NS tasks. Using these composite scores, paired-samples *t*-tests showed that there was a significant difference between the letter and object NS tasks on all measures, with longer spatial EVSs both inside [Figure 4A; *t*_(65)_ = 5.16, *p* < 0.001, *r* = 0.51, *d*_*z*_ = 0.64] and outside [Figure 4B; *t*_(65)_ = 5.22, *p* < 0.001, *r* = 0.66, *d*_*z*_ = 0.64] the ROIs and shorter temporal EVSs both inside [Figure 4C; *t*_(63)_ = −7.35, *p* < 0.001, *r* = 0.83, *d*_*z*_ = −0.92] and outside [Figure 4D; *t*_(63)_ = −7.91, *r* = 0.87, *d*_*z*_ = −0.99] the ROIs on the letter NS tasks.

When considering the task variations, for the letter NS tasks, paired-samples *t-*tests showed that none of the various manipulations produced statistically significant changes in either spatial EVS construct (Figures 4A,B; all *p*'s > 0.05), but that temporal EVS both inside and outside the ROIs (Figures 4C,D) was significantly higher on the VS and VPS tasks as compared to the LC task (all *p*'s < 0.05). However, for the object NS tasks, there was a significant difference in spatial EVS outside of the ROIs [Figure 4B; *t*_(66)_ = 5.31, *p* < 0.001, *r* = 0.75, *d*_*z*_ = 0.65], where participants had significantly longer EVSs on the OPS task as compared to the OC task.

We then examined the significant group by task interactions to determine how the effects of task composition and stimulus manipulations differed across development. First, to determine the contributions of the different developmental trajectories of letter and object NS tasks to the group by task interactions, we repeated the analyses of variance using the average letter and object composite scores. Significant interactions remained for both spatial and temporal EVS outside of the ROIs (all *p*'s < 0.05). These interactions may reflect the difference in the spatial EVS construct between letter and object NS tasks becoming more pronounced in the older age groups (Figure 4B), while the latter interaction may reflect a greater developmental decline for the temporal EVS construct for object NS tasks as compared to letter NS tasks (Figure 4D). When repeating the analyses of variance separately for the four letter NS tasks and the two object NS tasks, a significant group by task interaction only remained for temporal EVS outside of the ROIs on the letter NS tasks [Figure 4D; *F*_(9, 186)_ = 2.67, *p* < 0.01, $\eta_{p}^{2}$ = 0.11], possibly attributable to a decline in the effect of the VS and VPS manipulations with increasing age (Figure 4D).

### Relationships Between Behavioral Measures

We next explored the relationship between the various behavioral measures using the averaged *z*-scores for the letter and object NS tasks (Table 1). For the letter NS tasks, NS efficiency was positively correlated with both spatial EVS constructs and negatively correlated with all other measures for zero order correlations (all *p*'s < 0.05). Correlations for all measures, except spatial and temporal EVS within the ROIs (*p*'s > 0.05), remained significant when performing partial correlations with the effect of age removed (all *p*'s < 0.05).

**Table 1:** Correlations among NS measures.

	**1**.	**2**.	**3**.	**4**.	**5**.	**6**.	**7**.	**8**.	**9**.	**10**.
**LETTER NS TASKS**										
1. Efficiency	–	−0.67***	−0.47***	−0.48***	−0.58***	−0.64***	0.23	0.37**	−0.23	−0.26*
2. AT	−0.91**	–	0.75***	0.74***	0.65***	0.61***	−0.23	−0.34**	0.35**	0.35**
3. PT	−0.79**	0.87**	–	0.52***	0.86***	0.55***	−0.11	−0.17	0.34**	0.40**
4. FD	−0.78**	0.88**	0.77**	–	0.17	0.20	−0.36**	−0.39**	0.20	0.28*
5. SC	−0.86**	0.89**	0.94**	0.64**	–	0.73***	−0.01	−0.14	0.34**	0.36**
6. RC	−0.89**	0.88**	0.81**	0.67**	0.91**	–	−0.001	−0.12	0.32*	0.35**
7. sEVS ROI	0.38**	−0.42**	−0.24	−0.52**	−0.34**	−0.34**	–	0.88***	0.56***	0.50***
8. sEVS Other	0.43**	−0.47**	−0.26*	−0.55**	−0.42**	−0.41**	0.91**	–	0.43***	0.43***
9. tEVS ROI	−0.70**	0.70**	0.71**	0.61**	0.71**	0.61**	0.17	0.09	–	0.95***
10. tEVS Other	−0.71**	0.71**	0.74**	0.65**	0.72**	0.73**	0.15	0.11	0.98**	–
**OBJECT NS TASKS**										
1. Efficiency	–	0.43***	−0.60***	−0.13	−0.56***	−0.50***	0.04	0.25	−0.32*	−0.30*
2. AT	−0.82**	–	0.45***	0.31*	0.41**	0.34**	0.01	−0.12	0.28*	0.37**
3. PT	−0.90**	0.74**	–	0.28*	0.67***	0.59***	0.02	−0.09	0.63***	0.65***
4. FD	−0.55**	0.62**	0.62**	–	−0.39**	−0.08	−0.06	−0.15	0.13	0.16
5. SC	−0.89**	0.84**	0.90**	0.31*	–	0.64***	−0.02	−0.08	0.50***	0.50***
6. RC	−0.86**	0.80**	0.85**	0.42**	0.89**	–	−0.03	−0.18	0.40***	0.36***
7. sEVS ROI	−0.001	−0.15	0.10	−0.18	−0.12	−0.14	–	0.83***	0.36**	0.28*
8. sEVS Other	0.03	−0.18	0.11	−0.25	−0.12	−0.20	0.90**	–	0.32*	0.35**
9. tEVS ROI	−0.73**	0.69**	0.83**	0.45**	0.78**	0.73**	0.22	0.22	–	0.89***
10. tEVS Other	−0.75**	0.75**	0.83**	0.48**	0.80**	0.73**	0.15	0.22	0.94**	–

Zero order correlations are below the diagonal and partial correlations with age as a covariate are above the diagonal. NS, Naming Speed; AT, Articulation Time (ms); PT, Pause Time (ms); FD, Fixation Duration (ms); SC, Saccade Count; RC, Regression Count; sEVS, Spatial Eye-Voice Span; tEVS, Temporal Eye-Voice Span; ROI, Regions of Interest; Other, Outside ROI. N = 68. *p < 0.05; **p < 0.01; and ***p < 0.001.

For object NS tasks, NS efficiency was negatively correlated with all measures (all *p*'s < 0.05) except fixation duration and both spatial EVS constructs (all *p*'s > 0.05) for both zero order correlations and partial correlations with the effect of age removed (Table 1). The correlations among the letter NS measures were stronger than those for the object NS measures. Additionally, across both the letter and object NS tasks, the correlations between NS efficiency and the two spatial EVS constructs were weaker and frequently not statistically significant as compared to those observed between the other behavioral measures.

### Predicting Naming Speed Efficiency Across Development

To determine which NS component and eye movement variable best predicted letter and object NS efficiency and how these relationships changed across development, stepwise regression analyses were performed separately for each group (Table 2). Separate models were run for (a) NS components (articulation time and pause time), (b) eye movement variables (fixation duration, saccade count, regression count), and (c) spatial and temporal EVS. In each analysis, predictors were added if *p* < 0.05, and were dropped if *p* > 0.10. For the NS components, pause time significantly predicted letter and object NS efficiency in every age group, and the effect of articulation time grew from Grade 4 onwards. Whereas pause time became relatively less powerful with increasing age in predicting letter NS, it remained the stronger predictor at all ages for object NS.

**Table 2:** Stepwise regression analyses predicting NS efficiency.

**Letter NS efficiency**				**Object NS efficiency**			
**Group**	**Predictor**	**β**	***R*^**2**^**	**Group**	**Predictor**	**β**	***R*^**2**^**
**(A) NS COMPONENTS**							
Grade 2	Pause time	−0.92***	0.84***	Grade 2	Pause time	−0.92***	0.84***
Grade 4	Pause time	−0.74***	0.87**	Grade 4	Pause time	−1.07***	0.91**
	Articulation time	−0.38**			Articulation time	−0.45***	
Grade 7/8	Pause time	−0.41***	0.96***	Grade 7/8	Pause time	−0.81***	0.93***
	Articulation time	−0.62***			Articulation time	−0.40***	
Undergrads	Pause time	−0.39**	0.97**	Undergrads	Pause time	−0.82***	0.96***
	Articulation time	−0.61***			Articulation time	−0.48***	
**(B) EYE MOVEMENT MEASURES**							
Grade 2	Saccade count	−0.73***	0.96***	Grade 2	Saccade count	−0.74***	0.97***
	Fixation duration	−0.44***			Fixation duration	−0.59***	
Grade 4	Regression count	−0.81***	0.66***	Grade 4	Regression count	−0.84***	0.70***
Grade 7/8	Saccade count	−0.79***	0.93***	Grade 7/8	Saccade count	−1.02***	0.81***
	Fixation duration	−0.68***			Fixation duration	−0.85***	
Undergrads	Saccade count	−0.70***	0.93***	Undergrads	Saccade count	−1.16***	0.89***
	Fixation duration	−0.55***			Fixation duration	−0.70***	
**(C) EYE-VOICE SPAN CONSTRUCTS**							
Grade 2	–	–	–	Grade 2	tEVS ROI	−0.72**	0.52**
Grade 4	tEVS other	−0.72**	0.52**	Grade 4	–	–	–
Grade 7/8	–	–	–	Grade 7/8	tEVS ROI	−0.56*	0.31*
Undergrads	sEVS other	0.59**	0.46*	Undergrads	–	–	–
	tEVS ROI	−0.49*					

NS, Naming Speed; sEVS, Spatial Eye-Voice Span; tEVS, Temporal Eye-Voice Span. Grade 2: N = 13; Grade 4: N = 14; Grade 7/8: N = 21; Undergraduate: N = 20. *p < 0.05; **p < 0.01; and ***p < 0.001.

For the eye movement measures, saccade count and fixation duration both significantly predicted letter and object NS efficiency for all groups except Grade 4, with saccade count playing a larger role. In Grade 4, regression count was the sole significant predictor of both letter and object NS efficiency. This result may be due to minor variations in correlations or the limited sample size of this group. To test the pattern found in the other groups, saccade count, and fixation duration were forced as predictors in each Grade 4 analysis. Similar to the findings of the stepwise regression analyses, both saccade count and fixation duration significantly predicted letter and object NS efficiency (*R*^2^ = 0.79, *p* < 0.001 for letter NS efficiency and *R*^2^ = 0.90, *p* < 0.001 for object NS efficiency), with saccade count playing a larger role (β = 0.85, *p* < 0.001 vs. β = 0.41, *p* < 0.05 for letters, and β = 1.37, *p* < 0.001 vs. β = 1.07, *p* < 0.001 for objects, respectively).

For the EVS constructs, there were no clear trends with which constructs predicted letter NS efficiency. In Grade 4 students, temporal EVS outside the ROIs significantly predicted letter NS efficiency, while in undergraduate students, spatial EVS outside the ROIs and temporal EVS within the ROIs significantly predicted NS efficiency, with the former playing a greater role. No significant predictors were found for letter NS efficiency for the Grade 2 students or the Grade 7/8 students. Interestingly, temporal EVS within the ROIs significantly predicted object NS efficiency in both the Grade 2 and the Grade 7/8 students, diverging from the results observed for letter NS efficiency. None of the EVS constructs significantly predicted object NS efficiency in the Grade 4 students or the undergraduate students.

## Discussion

The goal of this study was to elucidate developmental trends in performance during NS tasks. Across the age groups assessed here, older participants performed better on NS tasks, characterized by higher NS efficiency and tighter temporal EVS, shorter pause and articulation times, shorter fixation durations, and fewer saccades and regressions. Across groups, participants were more efficient on the letter NS tasks than the object NS tasks. Both visual and phonological similarity influenced performance on NS tasks, indicating that both orthographic processing and, to a lesser extent, phonological processing are involved in NS performance, with a lower effect of phonological similarity in older groups. Finally, multiple regression analyses revealed important contributions of pause time, articulation time, fixation duration, and saccade count to the prediction of NS efficiency, with a notable decrease in the predictive power of pause time for letter NS efficiency accompanied by an increase in the predictive power of articulation time, in the older groups.

### Cross-Developmental Behavioral Trends in NS Performance

We found robust differences between groups on all behavioral measures; older groups were more efficient, had shorter fixation durations, articulation times, and pause times, and made fewer saccades and regressions than younger groups. These findings replicate previous behavioral studies using these tasks (e.g., Al Dahhan et al., 2014, 2017, 2020, in press). NS has been characterized as a microcosm of reading, involving the same cognitive processes that underlie reading and requiring them to occur in an automated fashion (Wolf and Bowers, 1999; Al Dahhan et al., 2020, in press). As such, NS has been found to be a strong predictor of reading ability (Kirby et al., 2003; Arnell et al., 2009; Al Dahhan et al., 2017). Thus, it is intuitive that as reading achievement improves across development, NS efficiency increases concurrently, likely linked to increased efficiency of the cognitive processing underlying reading (Figure 3A; Gordon et al., 2020). Shorter fixation durations, articulation times, and pause times indicate that encoding and processing alphanumeric and non-alphanumeric stimuli become more automatic across development, suggesting stronger orthographic processing and resulting in overall increased task efficiency (Rayner, 1997; Neuhaus et al., 2001; Bowers and Newby-Clark, 2002; Georgiou et al., 2008; Kirby et al., 2010; Araújo et al., 2011; Al Dahhan et al., 2017, 2020, in press). Of note, the number of saccades made during the NS tasks in this study, even in the undergraduate group, often exceeded the minimum number required to move the gaze sequentially from one item to the next. This may be because participants struggled to effectively control their eye movements during rapid task performance, or alternatively, because they scanned the matrix of items during the task as a consequence of a failure to completely devote their attentional resources to efficient task completion. As such, the decline in saccade count observed across age groups (Figure 3E) may reflect improvements in either of these oculomotor or attentional domains. Among the eye movement variables measured, saccade count was the strongest predictor of NS efficiency across development, indicating that oculomotor control and attention are likely very important in task performance. Regressions to previously named stimuli may reflect difficulties processing visual stimuli, representing insufficient information acquisition from an initial fixation, or alternatively, may be the result of oculomotor errors that require hypermetric saccades to be corrected (Rayner et al., 2006). Therefore, the developmental decline in regression count indicated by our findings (Figure 3F) may reflect improvements in cognitive efficiency or oculomotor control, consistent with the observed decreases in fixation duration and saccade count. Failure in automatizing these underlying cognitive processes at both the behavioral and brain levels has been indicated to underlie reading impairments found in readers with dyslexia (e.g., Al Dahhan et al., 2014, 2017, in press).

Skilled oral readers are able to begin visually inspecting and preparing responses for upcoming items while finishing the articulation of a previous item. Spatial EVS reflects the extent to which the eye is able to move ahead of the voice in such preparation, and is proposed to relate to readers' ability to update the phonological loop of their working memory with the identified names of upcoming items (Baddeley and Hitch, 1974; Laubrock and Kliegl, 2015). In the present study, we found no significant effect of group on spatial EVS, indicating that despite their presumably higher reading ability, older readers did not have higher spatial EVS than younger readers. This suggests that spatial EVS does not change significantly across the course of typical development. In apparent contrast with our findings, it has been previously shown that readers diagnosed with dyslexia present with shorter spatial EVS than typically developing readers (Pan et al., 2013). In synthesis with our findings, this suggests that an impaired ability to update the phonological working memory loop is not a universal phenomenon for all individuals that struggle with reading, such as the young and inexperienced, yet typical readers who participated in our study, but may instead be an intrinsic characteristic of individuals with reading disabilities such as dyslexia.

In contrast, we found a significant effect of group on temporal EVS, suggesting a developmental decline in temporal EVS that begins in Grade 4. Temporal EVS, or naming latency, reflects the time taken to complete all stages of cognitive and articulatory processing required to identify an item and prepare the articulation of its name (Jones et al., 2013). As such, our findings suggest that over the course of typical development, readers become more efficient at completing the stages of processing required for NS and reading, in keeping with the improvements in NS efficiency and pause time that were also observed.

### Comparison of Alphanumeric and Non-alphanumeric NS

Alphanumeric letter NS performance was more efficient than non-alphanumeric object NS performance (Figure 3A) and was associated with shorter articulations (Figure 3B) and pauses (Figure 3C), briefer fixations (Figure 3D), fewer saccades (Figure 3E) and regressions (Figure 3F), longer spatial EVSs (Figures 4A,B), and shorter temporal EVSs (Figures 4C,D), which are all indicators of improved NS task performance. More efficient performance on letter NS tasks may reflect participants' greater daily exposure to and dependence on letters for reading, as well as the higher visual complexity, greater set size, and potentially ambiguous names of the stimuli on the object NS tasks, which may slow cognitive processing and impair performance on these tasks. In addition, the shorter articulation time observed for the letter NS tasks is likely due to the lower number of phonemes in letter names as compared to object names. Of note, the longer articulation times for objects could reduce the amount of time available for pauses on the object NS tasks; as such, we may actually underestimate the comparatively higher pause times for object NS tasks, and by extension, the slower cognitive processing underlying object NS. Furthermore, our findings suggest that letter NS becomes progressively more efficient than object NS over the course of development (Figure 3A). This is consistent with previous findings that, while kindergarten students display no differences in alphanumeric and non-alphanumeric NS, alphanumeric NS becomes more efficient than non-alphanumeric NS by Grade 1 and continues to develop more rapidly beyond this point (Wolf et al., 1986). These behavioral results are further supported by neuroimaging findings of an increase of activation in the left-hemisphere reading network for letter NS tasks than object NS tasks, which suggests that the reading network is specific to letter stimuli (Al Dahhan et al., 2020, in press) and elucidates why alphanumeric NS is a greater predictor of reading than non-alphanumeric stimuli (Kirby et al., 2010).

### Effect of Stimulus Composition on Task Performance

Increasing the phonological and visual similarity of the stimuli both impaired NS task performance with respect to all constructs except spatial EVS, consistent with the proposed contributions of phonological processing (Torgesen et al., 1994, 1997) and orthographic processing (Bowers and Wolf, 1993; Bowers, 1995) to the NS-reading relationship. However, performance was impaired to a greater extent by visual similarity, suggesting that orthographic processing forms the bulk of the cognitive processing required for NS, as previously established (Compton, 2003; Al Dahhan et al., 2017, 2020, in press). Furthermore, the lack of an effect of increased phonological and/or visual similarity on spatial EVS suggests that typical readers were able to maintain a constant distance between the eye and the voice, and were able to consistently update their phonological working memory loop, despite local disturbances of increased task difficulty.

The effects of the phonological and visual manipulations on letter NS task performance were lower in the older groups, suggesting that the increased efficiency of the cognitive processing underlying reading may enable skilled readers to more effectively overcome phonological and orthographic challenges. There was a particular decline in the effect of phonological similarity over the course of development, with the phonologically similar manipulation of the letter NS task actually improving efficiency (Figure 3A) and decreasing saccade count (Figure 3E) in the undergraduate students, aligning with the shift from phonological processing-dependent phonetic reading to orthographic processing-dependent word-recognition reading across development (Ehri and McCormick, 1998).

Notably, while increased visual similarity increased articulation time, possibly reflecting residual cognitive processing after articulation onset that interferes with articulatory processes, increased phonological similarity decreased articulation time when combined with increased visual similarity or with object stimuli (Figure 3B). This may reflect priming of the articulation of phonologically similar words, increasing the efficiency of the execution of speech motor commands, and alleviating the detrimental effects of increased visual similarity and non-alphanumeric stimuli.

### Limitations

The findings of our study must be interpreted in the context of several limitations. While our findings provide evidence for developmental trends in NS performance, we acknowledge that these developmental trends are speculative due to the cross-sectional nature of this study and should be confirmed with longitudinal studies tracking typical readers from childhood to adulthood. Because our sample sizes for the Grade 2 and the Grade 4 groups were relatively smaller than the older groups, limited statistical power may have masked potentially significant effects of task manipulations in these younger groups. As such, non-significant effects of group and non-significant *post-hoc* between-groups contrasts for certain measures may not be true null effects and may be simply a result of low statistical power. This may be especially true for noisier measures, such as EVS, which may require high statistical power to detect significant differences between groups. In addition, measures of reading ability was not collected during this study because we were solely interested in the developmental trajectory of these NS tasks. Future studies should examine the degree to which the behavioral trajectory of these tasks is related to reading ability performance. Additionally, these smaller groups of younger readers may not be truly representative of the underlying populations, thus limiting the generalizability of our findings. Furthermore, while it provides correlational evidence regarding the cognitive processes underlying NS, this study cannot establish causal relationships due to its cross-sectional nature and did not include reading measures. Future longitudinal studies could evaluate these underlying cognitive skills and eye movement variables in young participants through non-alphanumeric tasks before reading instruction begins to determine the predictive ability of these skills in reading acquisition.

## Conclusions

This study revealed key developmental trends in NS performance, associated articulatory components and eye movement measures, the effects of alphanumeric and non-alphanumeric stimuli, and the contributions of phonological and orthographic processing to NS performance in healthy individuals. These findings provide insight into how the cognitive, articulatory, and oculomotor processes required for NS and reading, as well as the mechanisms that synchronize and coordinate them, evolve over the course of development. Additionally, this study provides novel insight into the coordination of eye movements and articulations by examining EVS, revealing that temporal EVS, a measure of overall processing efficiency, decreases over typical development, while spatial EVS, which reflects the efficiency of updates to the phonological working memory buffer, does not change significantly across typical development. This typical developmental trajectory could be compared with atypical developmental trajectories assembled for various clinical populations that experience reading difficulties. Observation of which NS tasks, articulatory components, and eye movement measures on which these clinical populations perform differently than typical readers will elucidate the exact nature of the cognitive dysfunction that underlies the reading difficulties experienced by these individuals. This will facilitate the development of targeted diagnostic tools to allow for early identification and more effective educational interventions to improve the literacy skills and life outcomes of individuals with reading disabilities.

## Data Availability Statement

The raw data supporting the conclusions of this article will be made available by the authors, without undue reservation.

## Ethics Statement

The studies involving human participants were reviewed and approved by Queen's University General Research Ethics Board. Written informed consent to participate in this study was provided by the participants' legal guardian/next of kin.

## Author Contributions

NA, JK, DB, and DM designed research. KE performed research. NA and KE analyzed data. NA, KE, JK, and DM wrote the paper. All authors contributed to the article and approved the submitted version.

## Conflict of Interest

The authors declare that the research was conducted in the absence of any commercial or financial relationships that could be construed as a potential conflict of interest.
